# The first complete mitochondrial genome sequence of an Australian raven (*Corvus coronoides)*

**DOI:** 10.1080/23802359.2017.1361365

**Published:** 2017-07-31

**Authors:** Subir Sarker, Karla Helbig, Shane R. Raidal

**Affiliations:** a Department of Physiology, Anatomy and Microbiology, School of Life Sciences, La Trobe University, Melbourne, VIC, Australia;; b School of Animal and Veterinary Sciences, Faculty of Science, Charles Sturt University, Bathurst, New South Wales, Australia

**Keywords:** Avian mtDNA, mitogenome phylogeny, family Corvidae, *Corvus coronoides*

## Abstract

Here, we report the complete mitochondrial genome of an Australian raven (*Corvus coronoides*). The mitogenome of *C. coronoides* was characterised as a circular molecule of 16,925 bp in length encoding a typically conserved structure similar to those of other *Corvidae.* It consisted of 13 protein-coding genes (PCGs), two rRNA genes, and 22 tRNA genes, with all protein-coding sequences commencing with methionine. The lengths of 12S ribosomal RNA and 16S ribosomal RNA were 980 bp and 1600 bp, respectively, and were located between tRNA-Phe and tRNA-Leu. The overall base composition of the mitogenome of *C. coronoides* was slightly higher AT (56.0%) content than GC (44.0%). A phylogenetic tree using available complete mitogenome sequences of the family *Corvidae* revealed a close evolutionary relationship of *C. coronoides* with the now extinct Chatham raven (*C. moriorum*), a large songbird that was native to the Chatham Islands east of New Zealand.

The Australian raven (*C. coronoides*) is a passerine bird in the family *Corvidae* native to southern and northeastern Australia. There are two clades of the genus *Corvus* recognised in Australia. A clade of Australian ravens, includes the Australian raven (*C. coronoides*), Little raven (*C. mellori*), and Forest raven (*C. tasmanicus*) whereas the clade of Australian crows (Scofield et al. 2017) includes the Torresian crow (*Corvus orru*) and Little crow (*Corvus bennetti*). All the recent genetic work on the evolution of the *C. coronoides* has been studied using morphological, and molecular characters dependent on the CO1, ND2, ND3, and CYTB (Jønsson et al. 2011). A well-resolved avian phylogenetic tree is required for understanding biogeographic evolutionary structure, whilst there are still major uncertainties in the position of many avian species due to a lack of abundant mitochondrial (mt) datasets.

The complete mitogenomes could play a major role to understand the origin, evolution and divergence time of speciation, as well as influencing conservation and management decisions (Eo et al. 2010). Here, we report a complete mitogenome of *C. coronoides* which will provide further insights into the species diversity, host phylogeny and ecological diversity of the Corvidae.

The blood sample used in this study was obtained from an Australian raven in the wild (year of sampling: 2015; GPS location: 35°21´24.92˝S, 149°13´20.182˝E), and stored in appropriate condition by the Veterinary Diagnostic Laboratory (VDL), Charles Sturt University under the accession number CS15-3763. Animal sampling was obtained in accordance with approved guidelines set by the Australian Code of Practice for the Care and Use of Animals for Scientific Purposes (NHMRC, 1997) and approved by the Charles Sturt University Animal Ethics Committee (Research Authority permit 09/046), and the total genomic DNA was extracted using an established protocol (Sarker et al. 2015; Sarker et al. 2016). The genomic library was prepared with an insert size of 150 paired-end. A HiSeq2500 sequencing platform (Illumina, Novogene, China) generated approximately 10.2 million sequence reads from the genomic DNA of Australian raven. The raw datasets were trimmed to pass the quality control based on PHRED score or per base sequence quality score, and the assembly of the mitochondrial genome was conducted according to the established pipeline in CLC Genomics workbench 9.5.4 under La Trobe University Genomics Platform (Sarker et al. 2017). Annotation was performed with MITOS (Bernt et al. 2013), and protein coding ORFs were further assessed using the CLC Genomics Workbench (version 9.5.4).

The complete mitochondrial genome sequence of *C. coronoides* had a circular genome of 16,925 bp (GenBank accession no. MF370524). It encoded 37 genes containing 13 protein-coding genes (PCGs), two rRNA genes, and 22 tRNA genes. The contents of A, T, C and G were 31.1%, 24.9%, 29.4% and 14.5, respectively. AT and GC contents of this mitochondrial genome were 56% and 44%, respectively. The proportion of coding sequences with a total length of 11,283 bp (66.66%), which encodes 3761 amino acids, and all protein-coding genes started with Met. The lengths of 12S ribosomal RNA and 16S ribosomal RNA were 980 bp and 1600 bp, respectively, and were located between tRNA-Phe and tRNA-Leu. The gene arrangement was similar to the complete mitochondrial genome of other *Corvidae* species.

Phylogenetic analysis was performed using complete mitochondrial genome sequence of a *C. coronoides* determined in this study with the other species belonging to the family *Corvidae* available in GenBank. The sequences were aligned using the MAFFT L-INS-i algorithm (Katoh et al. 2002), and the maximum likelihood (ML) tree with 1000 non-parametric bootstrap resamplings were generated using CLC Genomics workbench 9.5.4. The phylogenetic tree revealed that *C. coronoides* was clustered closely to Chatham raven (*C. moriorum),* which was one of the large songbird native to the New Zealand (Figure 1), and this finding is consistent with the previous research (Scofield et al. 2017). We concluded that the complete mitogenome of *C. coronoides* will be a useful database among the genus *Corvus* to study further host-phylogenetic relationship of *Corvidae* species, and suggest this may be an implication for the conservation of the species.

**Figure 1.**
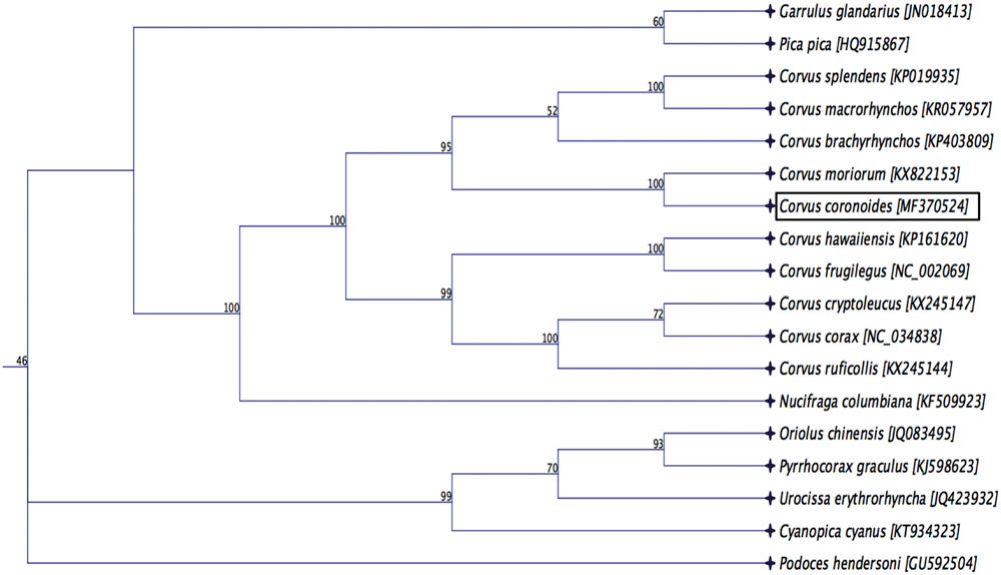
Maximum likelihood phylogenetic tree to infer host-phylogeny relationship among Corvidae family. ML-tree was constructed using complete mitochondrial genome sequences of the species belonging to the Corvidae family. The new complete mitochondrial genome of *C. coronoides* is highlighted by box.
